# Striatal Neurodegeneration that Mimics Huntington’s Disease Modifies GABA-induced Currents

**DOI:** 10.3390/brainsci8120217

**Published:** 2018-12-06

**Authors:** Jorge Flores-Hernández, Jeanette A. Garzón-Vázquez, Gustavo Hernández-Carballo, Elizabeth Nieto-Mendoza, Evelyn A. Ruíz-Luna, Elizabeth Hernández-Echeagaray

**Affiliations:** 1 Instituto de Fisiología, Benemérita Universidad Autónoma de Puebla, Puebla C.P.72570, México; jlvflores@hotmail.com (J.F.-H.); jeanettegarzon@gmail.com (J.A.G.-V.); l.gustavoh.carballo@gmail.com (G.H.-C.); evy_0927@hotmail.com (E.A.R.-L.); 2 Laboratorio de neurofisiología del desarrollo y la neurodegeneración, UBIMED, FES-Iztacala, Universidad Nacional Autónoma de México, México, FES-Iztacala, Av. de Los Barrios #1, Los Reyes Iztacala, Tlalnepantla C.P.54090, México; elynieto@gmail.com

**Keywords:** 3-NP, mitochondria, HD, gaboxadol, 2-AEMP, GABA_C._

## Abstract

Huntington’s Disease (HD) is a degenerative disease which produces cognitive and motor disturbances. Treatment with GABAergic agonists improves the behavior and activity of mitochondrial complexes in rodents treated with 3-nitropropionic acid to mimic HD symptomatology. Apparently, GABA receptors activity may protect striatal medium spiny neurons (MSNs) from excitotoxic damage. This study evaluates whether mitochondrial inhibition with 3-NP that mimics the early stages of HD, modifies the kinetics and pharmacology of GABA receptors in patch clamp recorded dissociated MSNs cells. The results show that MSNs from mice treated with 3-NP exhibited differences in GABA-induced dose-response currents and pharmacological responses that suggests the presence of GABA_C_ receptors in MSNs. Furthermore, there was a reduction in the effect of the GABA_C_ antagonist that demonstrates a lessening of this GABA receptor subtype activity as a result of mitochondria inhibition.

## 1. Introduction

Huntington´s Disease (HD) is a genetic neurodegenerative disorder originated by a gene mutation which results in the degeneration of neurons mainly in the nucleus striatum. The underlying mechanisms of degeneration are not fully understood, but experimental evidence suggests that the bioenergetics’ deficit produced by mitochondrial dysfunction and oxidative stress can be a significant factor in the development and progression of HD [1,2,3,4]. Indeed, the systemic administration of toxins or mitochondrial inhibitors, such as 3-nitropropionic acid (3-NP), produces movement disorders in primates and rodents [5,6,7,8,9] and cell damage that resembles the neuropathology observed in patients affected by HD [10,11,12]. This effect occurs because GABAergic striatal projection neurons (medium spiny neurons, MSNs) are particularly vulnerable to mitochondrial dysfunction and excitotoxicity [1,13]. For example, 3-NP in a concentration that produces 10% of inhibition of the succinate dehydrogenase (SDH) is capable of promoting the mitochondria permeability transition pore and stimulates Ca^2+^ increase, that in concert with the stimulation of glutamate receptors induces neuronal death of striatal MSNs [14,15]. The study of the physiological mechanisms responsible for the major vulnerability of MSNs may add to the development of therapeutic strategies, capable of lessening or stopping the neurodegenerative process and clinical manifestations. No cure for HD has been developed thus far; treatments are directed to attenuate symptomatology.

GABAergic transmission is one of the main objectives to develop treatments to reduce excitability increase at the striatum of HD patients, because GABA release produces phasic and tonic inhibition at the MSNs. Phasic inhibition is given by receptors located at the postsynaptic sites which are low sensitivity receptors, while tonic inhibition is given by high affinity receptors normally located at extra synaptic sites. GABA_A_ tonic mediated currents have been related with cellular protection in excitotoxicity. Therefore, tonic GABAergic inhibition has been proposed as a target for therapeutic management in HD [16,17,18].

Our group has demonstrated that 3-NP treatment can imitate early stages of HD [19] and alters GABAergic synapses and plasticity [20]. Also, the treatment with GABAergic agonists improves the behavior and activity of mitochondrial complexes in rodents treated with 3-NP, suggesting that GABAergic agonists exert a neuroprotective effect in instances of mitochondrial damage [21]. Additionally, GABAergic receptors undergo conformational changes that appear to protect MSNs from damage due to excitotoxicity [17]. To further the understanding of GABA receptors alterations in the early stages of HD, the objective of this study was to evaluate whether mitochondrial inhibition that mimics the early stages of HD modifies the kinetics of GABA-induced currents and pharmacology of GABAergic receptors in MSNs.

## 2. Material and Methods

All protocols and procedures employed in this study were reviewed and approved by the institutional board of bioethics (VIEP-2013-3557) and followed the national (NOM-062-ZOO-1999) and international guidelines of care and use on experimental animals.

### 2.1. Animals

Male C57BL/6 strain, 30-day-old mice were obtained from Harlan, Inc., México or ENVIGO RMS, México. The mice were housed in Plexiglas boxes at room temperature (24–26 °C) under a 12:12 h light/dark cycle with free access to food and water, and assigned to a group treated with 3-NP (15 mg/kg, diluted in phosphate buffer (0.1 M PB, pH 7.4, dissolved in 1 mL/20 g), i.p. over 5 days) or to a control group that only received the pharmaceutical excipient (0.1 M PB, pH 7.4, 1 mL/20 g) in an equal amount. 2 days after last treatment injection, the electrophysiological recordings were carried out [19].

### 2.2. Reagents

Unless otherwise stated all reagents were purchased from Sigma-Aldrich (St. Louis, MO, USA).

### 2.3. Slice Preparation

Tissue slicing and separation were performed according to previously described methods [22,23]. Dissection of the striatum was limited to the region that was rostral and dorsal to the anterior commissure to reduce contamination by the globus pallidus.

Slices were maintained between 1 and 6 hours at room temperature (20–22 °C) in Earle’s balanced salt solution (EBSS), buffered with sodium bicarbonate (NaHCO_3_), supplemented with 1 mM pyruvic acid, 0.005 mM glutathione, 0.1 mM N^G^-nitro-L-arginine and 1 mM kynurenic acid, and bubbled with 95% O_2_/5% CO_2_. The pH was adjusted to 7.4 with NaOH and osmolarity adjusted to 300 mOsm/L.

After at least 1 hour of incubation, slices from the striate nucleus were prepared for enzymatic treatment. Each slice was placed in a culture chamber containing 40 mL of Hank’s balanced salt solution (HBSS) mixed with 0.2 mg papain (Calbiochem, San Diego CA, USA), buffered with 4-(2-hydroxyethyl)-1-piperazineëthanesulfonic acid (HEPES), bubbled with O_2_ and maintained at 35 °C for 10 min. The solution was supplemented in the same way as the EBSS.

Subsequent to enzymatic digestion, the tissue was washed with a solution of isethionate and later mechanically separated with various sizes of flame-polished Pasteur pipettes. Suspensions of cells were seeded in 35-mm polystyrene Petri dishes (Nunclon Surface, NUNC, Rochester, NY, USA) mounted on the recording chamber coupled to a microscope. After 10 min of incubation, the suspension was washed with a solution containing 140 mM NaCl, 23 mM glucose, 15 mM HEPES, 2 mM KCl, 2 mM MgCl_2_, 1 mM CaCl_2_ and 1% phenol red, bubbled with O_2_ pH was adjusted to 7.4 with NaOH and osmolarity adjusted to 300 mOsm/L to prepare the tissue for subsequent recording using the voltage-clamp technique.

### 2.4. Whole-Cell Patch-Clamp Technique

Whole-cell voltage-clamp was used to record GABA-induced currents in neurons of the striate nucleus. Recording electrodes were pulled from borosilicate capillary tubes (1B120F-4, WPI, Sarasota, Florida, USA) with a micropipette puller (P-97, Sutter Instruments, CO, USA) and a resistance ranging from 4 to 8 MΩ.

The internal solution consisted of 175 mM N-methyl-D-glutamine (NMDG), 40 mM HEPES, 2 mM MgCl_2_, 10 mM ethylene glycol-bis (β-aminoethyl ether)-N, N, N’, N’- tetra acetic acid (EGTA), 12 mM phosphocreatine, 3 mM Na_2_ATP, 0.35 mM Na_3_GTP and 0.1 mM leupeptin, adjusted to a pH of 7.3 with H_2_SO_4 /_ NMDG and 265-270 mOsm/L. The external solution consisted of 127 mM NaCl, 20 mM CsCl, 5 mM BaCl_2_, 2 mM CaCl_2_, 12 mM glucose and 10 mM HEPES, adjusted to a pH of 7.4 with 300–305 mOsm/L NaOH and was supplemented as appropriate with GABAergic agents. Recordings were obtained with a Multiclamp 700A voltage clamp amplifier (Molecular Devices, Foster City, CA), controlled with pCLAMP version 8 (Molecular Devices, Foster City, CA) run on a Pentium (IV, 3.20 GHz) computer with a Digidata 1322A interface (Molecular Devices, Foster City, CA).

Once the seal was broken, only cells with an input resistance (R_IN_) less than 25 MΩ were included in the study. To record GABA-induced currents the holding potential was set at 0 mV, allowing access to the chlorine-based GABA-activated current, whose equilibrium potential is about −80 mV. Additionally, maintaining the membrane continuously at 0 mV inactivates sodium and calcium currents. Potassium was blocked by the Cs^+^ and Ba^2+^ present in the external solution.

### 2.5. Drug Application

**Figure 1:**
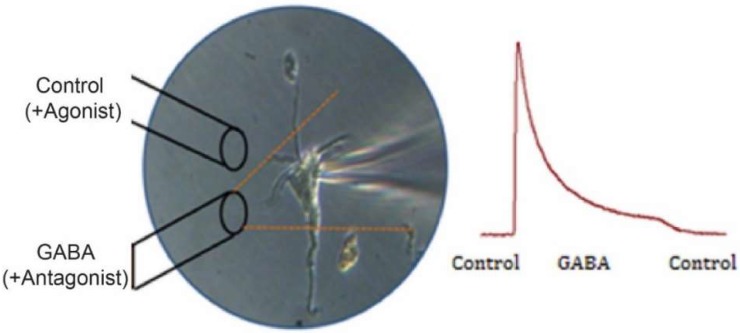
Application of solutions using capillaries. The application of solutions was controlled using solenoid valves; on the right of the photo, there is a representative trace of the current generated by the GABA application.

GABA-activated currents (I_GABA_) were induced by applying the compound using a system of capillaries placed at 45° with respect to each other and at a distance of 200 to 600 µm from the recorded cell. One capillary contained the external solution alone (control), and the other had the external solution and 1 of 2 ligands (Figure 1). The solution was changed using solenoid valves (The Lee Company, Essex CO LFAA 1201718H) controlled by the output of the Digidata 1322A system (Molecular Devices, Foster City, CA) and a control apparatus designed in our laboratory. The ligands used were 4, 5, 6, 7-tetrahydroisoxazolo (5,4-c) pyridin-3-ol (THIP, Gaboxadol), which is an agonist of GABA_A_ receptors that contain the δ subunit but an antagonist of GABA_C_ receptors, the negative allosteric modulator L-655,708 (TOCRIS Bioscience, Bristol, UK) an inverse agonist of GABA_A_ receptors containing the α_5_ subunit, as well as the GABA_C_ antagonist 2-Aminoethyl methylphosphonate (2-AEMP, this compound was a gift from Dr. Lobo, University of Maryland, USA).

### 2.6. Electrophysiology Protocols

**Application of GABA**: With a membrane holding potential of 0 mV, GABA was applied for 5 s; the entire protocol lasted 90 s.

**Recovery protocol:** This protocol was used to study the time constant for the recovery of the current to 100% after the initial application. A test pulse was delivered, followed by a quiet period increased in steps of 5 s, at which point a second pulse was delivered. The interval between the initial pulses was 90 s. Each pulse used a holding potential of 0 mV.

**Current versus voltage (IV) protocol:** Square voltage pulses were applied in steps of 20 mV to achieve a holding potential of −80 mV. During the pulse, GABA was applied for 5 s. The protocol lasted for 90 s.

**Control condition:** The cell was continuously perfused with the external solution using capillary 1 for the duration of the protocol, which was interrupted for 5 s to bathe the cell with 0.1, 0.3, 1, 3, 10, 30, 100, 300 or 1000 µM GABA through capillary 2. This experimental condition was used as the control and for the wash out to eliminate the effect of the application of the GABA antagonists.

**GABA + antagonist condition**: The bath was maintained with the external solution and interrupted with a cell bath with a solution for the co-application of GABA and Gaboxadol, GABA and L-655,708, or GABA and 2-AEMP.

### 2.7. Statistical Analysis

The antagonistic effect was measured as the reduction in the amplitude of the peak of the I_GABA_ with respect to the control and wash conditions. The percent reduction was calculated as follows:

% reduction=((δINE/((δIC+δIL)/2))−1)∗100

where *δIC* is the GABA induced current density in the control condition, *δIL* is the GABA induced current density in the wash condition, and *δINE* is the GABA induced current density in the presence of the GABA antagonist. Values are reported as the mean ± standard error of the mean (SEM). Curve fits and graphs were generated in Origin 9.1 (Microcal Software Inc., North Hampton, MA). Data analysis was performed using analysis of variance (ANOVA) followed by a multiple comparison test with the significance set at *p* < 0.05.

## 3. Results

A total of 173 cells were recorded: 150 MSNs and 23 giant interneurons. Half of the MSNs belonged to the control group and half to the group from mice treated with 3-NP. Only recorded neurons with a membrane resistance ≥1 GΩ and an input resistance <25 MΩ were included in the sample.

The size of the cells was determined by measuring the cell capacitance and using a capacitance/area ratio of 1 µF/cm^2^. 95% and 98% of the neural population were MSNs whose capacitance ranged from 3 to 6 pF (Figure S1). There was no difference in the measured capacitance in the cells of the 2 groups (Figure S1).

In each experiment, cellular viability was evaluated with a voltage ramp (from −100 mV to +40 mV) lasting 300 ms to generate Na^+^ (I_Na_^+^ < 1 nA) and Ca^2+^ (I_Ca_^2+^ < 100 pA) currents. Cells with values lower than these were excluded from analysis.

### 3.1. Recording I_GABA_

#### Equilibrium Potential for I_GABA_

The current versus voltage (IV) protocol was used to generate the I_GABA_ (100 µM) at different voltages (Figure S2A,B). To determine the equilibrium potential for I_GABA_, a second-order polynomial was fit to the peak current-voltage data (Figure S2C,D). The equilibrium potential was almost the same for both experimental groups, but the peak current and current density was greater for the control group than for the 3-NP group evaluated at 0 mV but these differences were not statistically significant.

### 3.2. GABA-Activated Currents (I_GABA_)

**Figure 2:**
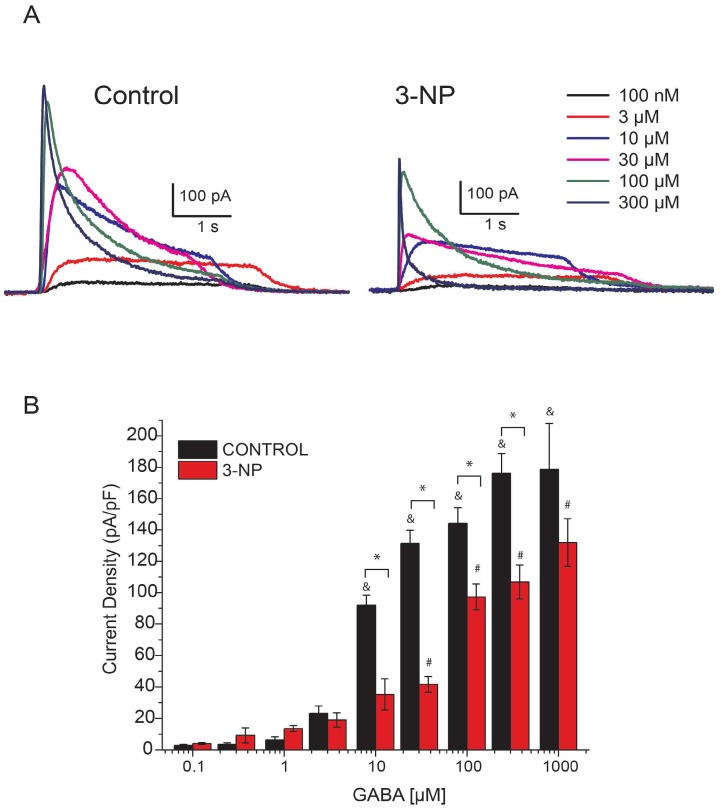
Dose-dependent response of GABA-activated current. (**A**) Representative traces of the activated current for 0.1, 3, 10, 30, 100, and 300 µM GABA from the MSNs of control and 3-NP mice. (**B**) Concentration dependent response to GABA is expressed as current density. The response to 10, 30, 100, and 300 µM of GABA was statistically different between control and 3-NP groups (F_1, 94_ = 6.39, *p* < 0.01, two way ANOVA), the GABA induced current amplitude increased significantly in both groups by rising the concentration of GABA from 10 µM for control group and from 30 µM for 3-NP groups (F_8, 94_ = 26.02, *p* < 0.05, two way ANOVA). Significant differences among GABA concentration are indicated with (^&^) for control group and with (^#^) for 3-NP groups and with a (*) for differences between 3-NP and control groups. Control group (black), 3-NP group (red). ^&, #,^ * *p* < 0.05.

Given the changes in membrane capacitance, I_GABA_ was normalized to obtain the peak current density as pA/pF. I_GABA_ was evoked using different concentrations of GABA (0.1, 0.3, 1, 3, 10, 30, 100, 300 and 1000 µM); the resulting plots of current density as a function of GABA concentration are shown in Figure 2A.

The peak density of I_GABA_ in neurons of animals from the 3-NP group was compared with that of the control group (Figure 2B).

#### GABA Dose-Response Curve in Medium Spiny Neurons

**Figure 3:**
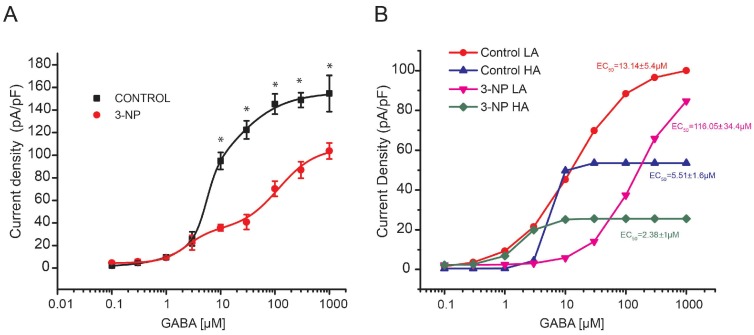
GABA dose-response curves. (**A**) GABA dependent concentrations response to 0.1 µM (*n* = 12), 0.3 µM (*n* = 10), 1 µM (*n* = 10), 3 µM (*n* = 12), 10 µM (*n* = 12), 30 µM (*n* = 12), 100 µM (*n* = 15), 300 µM (*n* = 15) and 1000 µM (*n* = 15) in both experimental groups * *p* < 0.01. (**B**) Peeling of sigmoidal functions plots to identify 2 different populations in the control group (LA EC_50_ = 13.14 ± 5.478, and HA EC_50_ = 5.51 ± 1.695, F_7,2_ = 1195.24, *p* = 0.0001, double logistic regression) and in the 3-NP group (LA EC_50_ = 116.05 ± 34.4 and HA EC_50_ = 2.38 ± 1.09, F_7,2_ = 248.69, *p* = 0.004, double logistic regression).

Pharmacological effects on a population of receptors can be reflected in dose-response curves. To determine the EC_50_ for GABA, a double-logistic dose-response curve was generated, resulting in 2 EC_50_ values for each group. In the control group, EC_50_ 1 was 13.14 ± 5.4 µM and EC_50_ 2 was 5.51 ± 1.6; in the 3-NP group, EC_50_ 1 was 116.05 ± 34.4 µM and EC_50_ 2 was 2.38 ± 1.0 (Figure 3B). These results suggest the existence of more than 1 population of receptors with different pharmacological sensitivities, where the effects of the same compound on the different populations are added. The 2 populations with different EC_50_ values are observed in the dose-response curves as different phases associated with each sigmoidal component.

Knowing the parameters for the pharmacological activities for each population with respect to the same compound, it is possible to separate 1 population from the other. We developed algorithms to differentiate the distinct parameters of the summed sigmoidal functions in Origin. This procedure is called “peeling of sigmoidal functions” and helps to distinguish the population of receptors with a low affinity for GABA (LA) from the population with a high affinity (HA) in the 2 experimental groups (Figure 3B). The EC_50_ of the population with high affinity to GABA was not different between the 3-NP and control groups, but a difference was found in the low-affinity populations between the 2 experimental groups (Figure 3B).

### 3.3. GABA Dose-Response Curves in Giant Interneurons

Recordings from cholinergic interneurons did not show differences in the current density versus GABA concentration used between the 2 experimental groups (Figure S3). These results demonstrate that cholinergic interneurons are not affected by mitochondrial inhibition of this model of HD.

### 3.4. Kinetics of the I_GABA_

The following parameters were analyzed to study the kinetics of GABA_A_ receptors: steady state/peak current, time to peak and time constant (tau) of desensitization.

#### 3.4.1. Steady-State (SS)/Peak Current

**Figure 4:**
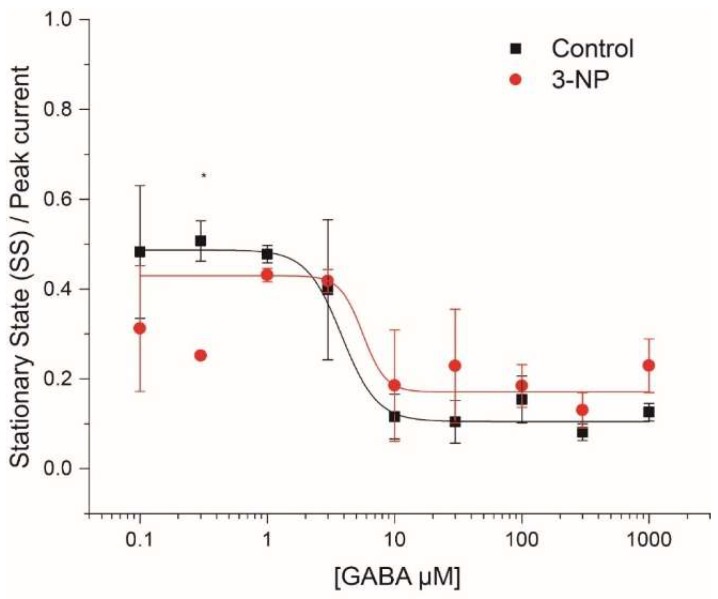
Steady-state versus peak GABA-activated current. The SS/peak parameter was used as a measure of GABA receptor desensitization for concentrations of 0.1 µM, 0.3 µM, 1 µM, 3 µM, 10 µM, 30 µM, 100 µM, 300 µM and 1000 µM in MSNs of both evaluated groups. Data Analysis showed differences in 0.3 µM GABA concentration but no between groups or in the interaction (F_8, 17_ = 5.53, *p* < 0.001, Two way ANOVA).

The steady-state to peak current ratio ranges between 0 and 1, as shown in Figure 4. From the dose-response curve, the steady state/peak current ratio shows that at low GABA concentrations (<10 µM), currents generated present a desensitization of approximately 50%, while at higher doses (>10 µM), the desensitization is approximately 95%. But the groups did not present statistical differences.

#### 3.4.2. Time to Peak

**Figure 5:**
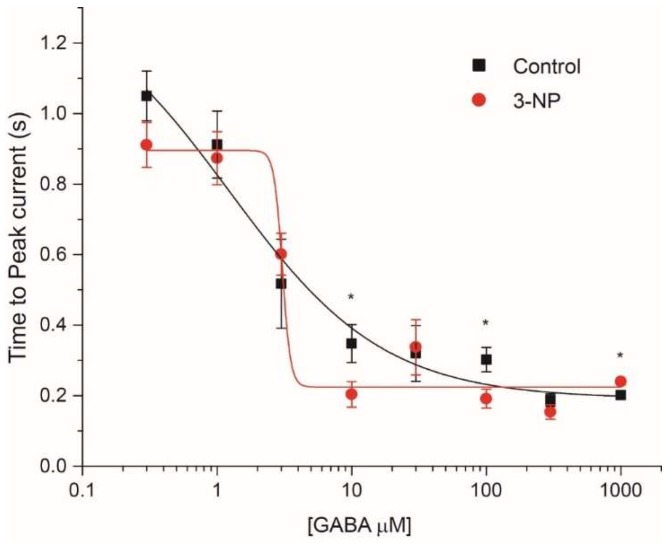
Time to peak of the GABA current. The activation time of I_GABA_ was different only for GABA concentrations of 10 µM, 100 µM, and 1000 µM (F _7, 15_ =69.36, *p* < 0.05, Two ways ANOVA).* *p* < 0.05.

The time to peak was used as a measure of the activation time of I_GABA_. The values obtained at GABA concentrations of 10 µM, 100 µM and 1000 µM were significantly faster than those obtained with 0.1 µM, 0.3 µM, and 3 µM of GABA but we did not find significant differences between the control and the 3-NP groups except for 10 µM, 100 µM and 1000 µM (Figure 5).

#### 3.4.3. Time Constant of Desensitization (tau,τ)

The speed and desensitization of the GABA_A_ receptor depend on the composition of the subunits. In our experiments we only identified significant differences in the values of τ between experimental groups for the GABA concentration of 0.1 µM (Table 1).

### 3.5. Recovery of I_GABA_

**Figure 6:**
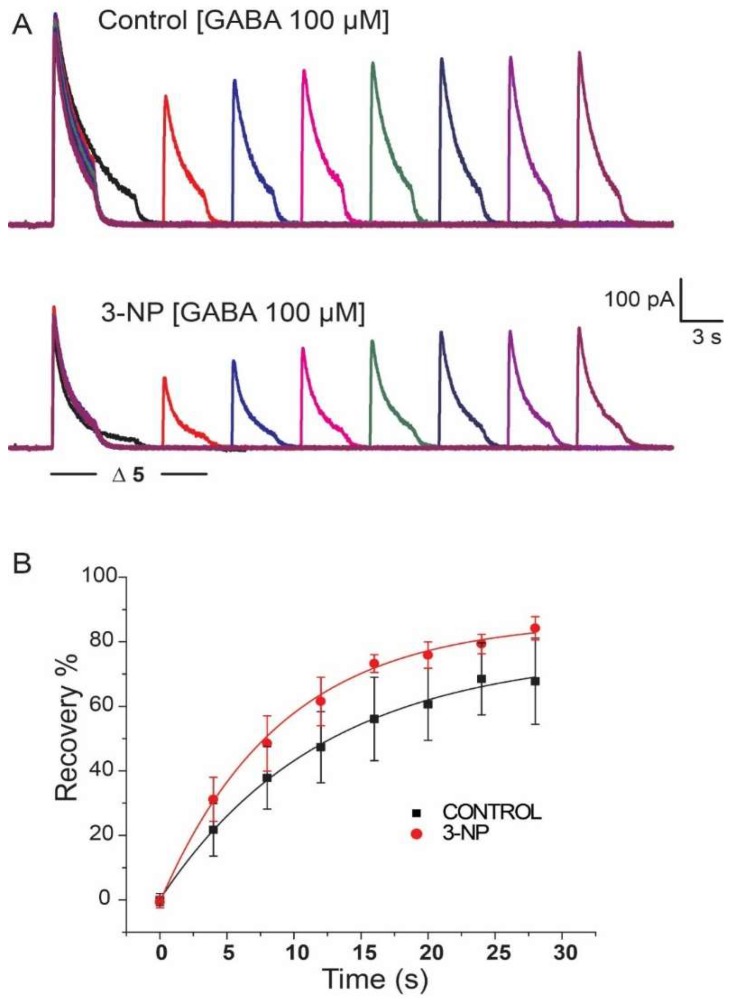
Recovery of I_GABA_. (**A**) Representative plot of the GABA (100 µM) recovery protocol for a control group neuron and for a 3-NP group neuron. (**B**) Plot of the percentage of current recovery versus time for the application of 100 µM GABA. Recovery time for the control group was similar for the recovery time for the 3-NP group.

The recovery protocol described in the methodology section was used to evaluate both experimental groups. Figure 6A illustrates traces obtained for the control and 3-NP groups. The percentage of recovery of the current was plotted versus time and was fit to a first-order decaying exponential, yielding values similar for the control group and for the 3-NP group (Figure 6B).

Given the characteristics observed in the I_GABA_ dose-response, we questioned the role of high-affinity and low-desensitization receptors in MSNs of our HD animal model. GABA extra synaptic receptors are known to be high affinity receptors which are sensitive to ischemic alterations and may change in degenerative disorders, and then we decided to evaluate GABA receptors with the α5 and δ subunits.

### 3.6. Effects of the α5 Subunit Inverse Agonist L-655708

**Figure 7:**
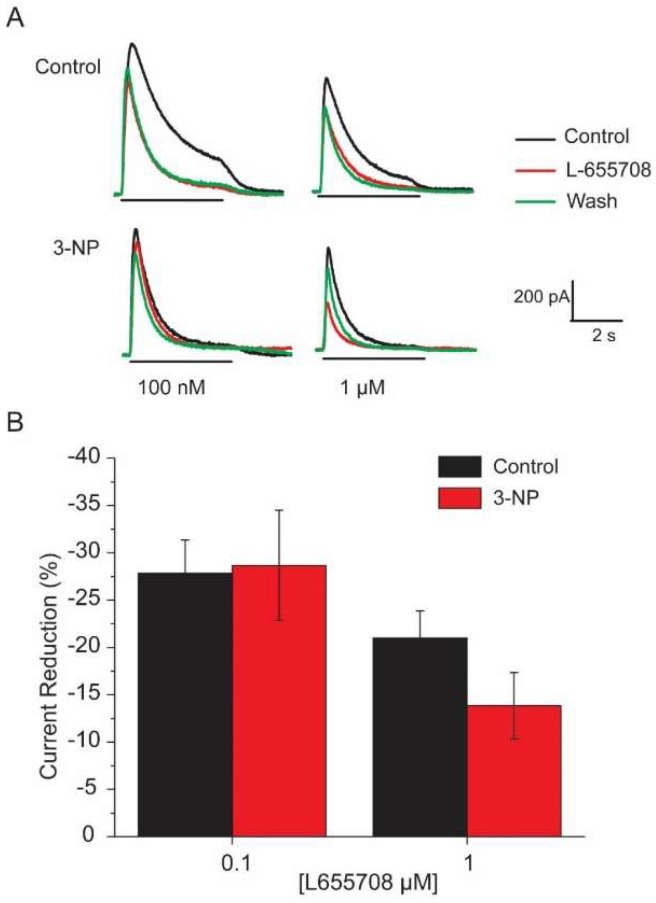
L-655708 effects on GABA induced current. (**A**) Representative traces of the current generated by GABA in the presence of 100 nM and 1 µM L-655708 in the MSNs of the control and 3-NP groups. (**B**) Plot of the GABA induced current reduction in the presence of two different concentrations of L-655708.

The percent reduction in I_GABA_ induced by 100 µM GABA was determined in the MSNs in the presence of 3, 10, 30, 100 and 1000 nM L-655708, a GABA_A_ receptor α5 subunit inverse agonist (Figure 7). The dose-response analysis of the GABA current density in the presence of L-655708 resulted in an EC_50_ of 119.11 ± 52.8 for the control group and EC_50_ of 95.85 ± 43.02 µM for the 3-NP group. Figure 7A shows representative traces of the effect of L-655708 100nM and 1µM on the GABA_A_ receptors in control and in the 3-NP group. Figure 7B shows plots of the current density of the experiments with the L-655708. Note that no significant differences were found in the percent reduction of the current due to the co-application of L-655708 and 100 µM GABA between the 2 groups. These results demonstrate that GABA receptors with the α5 subunit are not altered as a result of the damage produced by mitochondria inhibition.

### 3.7. Effects of the Gaboxadol

**Figure 8:**
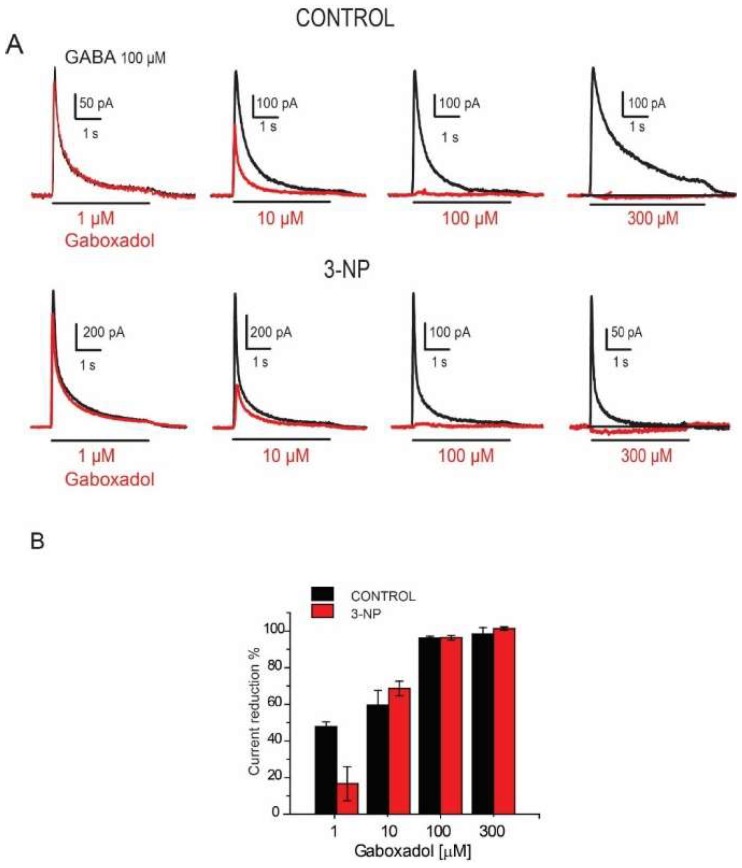
Gaboxadol effect on GABA induced current. (**A**) Representative traces of the GABA induced current (100 µM) with a co-application of Gaboxadol in 1 μM, 10 μM, 100 μM, 300 μM in both experimental groups. (**B**) Plot of the GABA induced current reduction recorded from the MSNs using increasing concentrations of Gaboxadol in control and 3-NP groups.

The effects of Gaboxadol, an agonist of GABA_A_ receptors with δ subunits and an antagonist of GABA_C_ receptors, was evaluated in the presence of 100 µM GABA. Figure 8A shows representative traces of these effects. Surprisingly, we observed a concentration-dependent reduction in I_GABA i_n both evaluated groups instead of the expected increase.

The percent reduction in current in the MSNs was analyzed at 1, 10, 100 and 300 µM of Gaboxadol giving an EC_50_ = 15.67 ± 1.968 for control neurons and EC_50_ = 9.81 ± 3.562 for neurons of the 3-NP group. The statistical analysis did not show significant differences between neurons in the control group versus those in the 3-NP group (Figure 8B).

### 3.8. GABA_C_ Receptors Reduction in 3-NP Group

**Figure 9:**
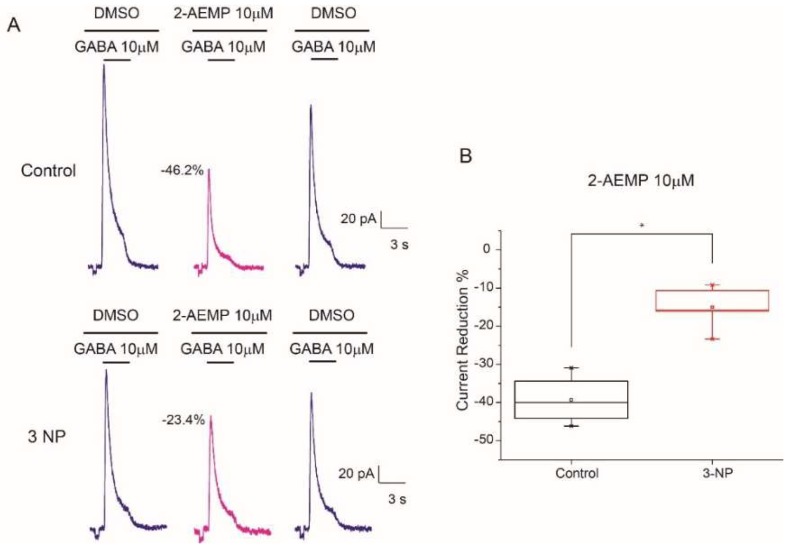
2-AEMP effect on GABA induced current. (**A**) Representative traces of the GABA induced current (10 µM) with a co-application of 2-AEMP (10 µM) in Control (top) and 3-NP groups (bottom). GABA administration was co-applied with DMSO used as an excipient for 2-AEMP. (**B**) Box plots illustrating the current peak reduction of I_GABA_ induced by the GABA_C_ antagonist. MSNs from the 3-NP group were significantly less inhibited by the 2-AEMP compared to control neurons (t_7_ = −6.015, *p* < 0.001).

Pharmacological inhibition of GABA induced currents by Gaboxadol suggested the presence of GABA_C_ receptors, since the Gaboxadol may act on GABA_C_ and GABA_A_ receptors we opted for the specific evaluation of GABA_C_ function in striatal MSNs. To do that evaluation, we used the 2-Aminoethyl methylphosphonate trifluoroacetate salt (2-AEMP) a competitive GABA_C_ antagonist. To see the effect of the 2-AEMP on high affinity GABA receptors we used a GABA concentration of 10 µM as reported by other studies [24]. 2-AEMP reduced I_GABA_ peak current in neurons of both; control and 3-NP-treated mice (Figure 9A); however, the reduction in the 3-NP group was significantly smaller (−15.03 % ± 2.49) than in the control group (−39.29 % ± 2.49), suggesting that GABA_C_ receptors are reduced as a result of mitochondrial inhibition (Figure 9B).

## 4. Discussion

The aim of this study was to evaluate whether mitochondrial inhibition that mimics early stages of HD modifies the kinetics of GABA-induced currents and pharmacology of GABAergic receptors in MSNs.

Our data showed that cells from the mice treated with 3-NP exhibited a reduction in the GABA gated peak current in concentrations of 10, 30, 100, 300 and 1000 μM. High affinity receptors did not show significant differences in the peeling of sigmoidal functions analysis. Differences were observed in the high affinity receptors as observed with concentrations higher to 10 μM of GABA. No changes were observed in the stationary state and in the time to peak in most of the evaluated concentrations. The recovery of GABA current neither exhibited significant changes between groups.

We tested an antagonist (L655708) and agonist (Gaboxadol) of GABA receptors containing the α5 subunit which has been implicated in GABA transmission neuroprotection. The exploration of high affinity receptors with the α5 antagonist L-655708 did not present differences between groups and exhibited a small reduction of the I_GABA_ amplitude. The evaluation of Gaboxadol did not present an increase in the I_GABA_ amplitude. On the contrary, a reduction in the I_GABA_ amplitude was observed in a dose concentration mode. The effect of Gaboxadol on GABA_A_ receptors varies depending on GABA subunit composition. It is a partial agonist of GABA receptors with α1β2γ2 and full agonist GABA receptors with α5 subunit, however is an antagonist of GABA_C_ receptors containing the *ρ*1 subunit [25,26,27,28]. To verify the presence of GABA_C_ in dissociated MSNs and evaluate the role of those receptors, some experiments were carried out with the competitive GABA_C_ antagonist 2-AEMP, this compound reduced GABA gated current in less proportion than it did in control cells, demonstrating that these receptors were reduced as a result of mitochondria inhibition.

### 4.1. MSN Capacitance Decreases as a Result of 3-NP

The recorded neurons were identified by their morphologies, capacitances and input resistances. The capacitance of the MSNs did not significantly decrease in the group treated with 3-NP. Although the reduction in capacitance in dissociated cells is less evident than in recordings from cells in slice preparations. Our lab has demonstrated that the MSNs of mice treated with 3-NP in same concentration of this study, have a smaller number of spines and narrower dendrites [29]. These changes reduce the capacitance, a result also found in other animal models of HD [30,31].

### 4.2. Recording I_GABA_

The equilibrium potential for GABA was the same between the 2 groups, as well as the recovery time of the response to 100 µM GABA. Interestingly, the current density was greater for neurons treated with 3-NP at the lowest GABA concentration (0.1 to 1 μM), indicating that GABA high affinity receptors increased in these group of cells, probably as compensatory response in the early stages of striatal neurodegeneration [17].

Analysis of the dose-response curve in the MSNs showed the presence of at least 2 populations of GABAergic receptors exhibiting different affinities for and efficacies in response to GABA. MSNs treated with 3-NP showed a change in EC_50_ of a population of receptors with low affinity, suggesting that mitochondrial inhibition modifies the expression and/or composition of the subunits of the GABA_A_ receptor. There is experimental evidence of changes in the subunits of GABA receptors in a number of neuropathologies and in hypoxic events [32].

Administration of GABA at low concentrations (<10 µM) resulted in little desensitization in the control group and 3-NP group. At higher concentrations (>10 µM), both groups showed a desensitization of 95% of the current with no significant differences between the groups.

The activation time was not different between groups. Analysis of the kinetics revealed one-time constants of desensitization (τ_1_) that was not significantly different for all GABA concentrations. Both, the speed and degree of desensitization depend on the subunit composition and they may change [33,34,35]. However, the lack of kinetics differences in our experimental groups was due to the fact that our animal model mimics early stages of HD and GABA responses are not damaged as they may be in advanced stages of the illness.

The giant interneurons did not show any changes due to treatment with 3-NP, neither in their capacitance nor in the kinetics of the GABA current, similar to the behavior of neurons in HD-transgenic mice [36,37]. It is not known how interneurons remain the same despite the observed changes in the spiny projection neurons in advanced stages of the illness in HD patients and animal models.

### 4.3. Effect of L-655708

We evaluated whether high affinity GABA receptors containing α5 subunit, that have been involved in neuroprotection were present in the MSNs. Our data showed that they are present but did not show significant differences in the reduction percentage of the GABA current when 100mM of GABA was co-applied to at different concentrations of the specific antagonist L-655708 [38], suggesting that expression of the α_5_ subunit of the GABA_A_ receptor is not altered by treatment with 3-NP, as it happened in the R6/1 mouse model of HD at 6 months [18].

Because the experiments were performed in the soma of the dissociated MSNs, we cannot rule out the possibility of a change in the expression of the receptors in the dendrites. L-655708 is 50-100 times more selective for the GABA_A_ receptors containing the α_5_ subunit than for those that contain α_1_, α_2_, α_3_ and α_6_ and they are located mainly at extrasynaptic sites [39].

### 4.4. Effect of Gaboxadol

Gaboxadol is a partial agonist of GABA_A_ receptors that express the α_4_ and δ subunits full agonist of GABA receptors containing the α_5_ [25,26,40]; but it acts as an antagonist of GABA_C_ receptors which contain rho subunits [27]. We did not observe any agonistic effect of Gaboxadol when recording from the soma of dissociated MSNs. Gaboxadol acted as an antagonist to the I_GABA_ in both the control cells and those from animals in the 3-NP group, indicating that this effect could occur through actions on the GABA_C_ receptors of the MSNs; these receptors are in the striatum [41], and may be able to form heteromers with GABA_A_ in the striatum as occurs in other regions [42,43]. Still our results were unexpected, because in MSNs GABA_A_ mainly have been described [22,23]. The experiments with the 2-AEMP were conclusive to demonstrate that GABA_C_ are in MSNs and they are reduced in 3-NP tissue. Indeed, a reduction in GABA*ρ*3 subunit has also been documented in theR6/2 transgenic mice model of HD [44], supporting that GABA_C_ receptors are affected in HD pathology.

GABA_C_ receptors have high affinity and slow desensitization to GABA and may mediate GABA responses to low GABA concentrations. They are thought to be located at perisynaptic sites and could participate in the tonic inhibition of GABA suppressing neuronal excitability, which in the presence of 3-NP and HD is increased [8].

## 5. Conclusions

Little is known of the long-term behavior and of the changes that occur in the GABAergic synapses in striatal degeneration induced by mitochondrial dysfunction. Evidence suggests that adaptive changes occur in synaptic function, and some of them can be protective to counteract the pathology [45,46]. Our data showed that GABA_C_ receptors exhibited a reduction in its function on striatal cells as a result of the mitochondria inhibition. Thus, further experiments should address the pharmacological profile and function of GABA_C_ receptors in the projection neurons at the striatum and in behavioral evaluations. Moreover, this information invites us to explore GABAergic function in depth in particular GABA_C_ receptors as a therapeutic target in striatal neurodegeneration produced by mitochondria dysfunction in HD and will help in the treatment of patients afflicted by HD.

## Figures and Tables

**Table 1:** Time constant of desensitization.

	Control		3-NP	
GABA µM	τ ms	SE	τ ms	SE
0.1	975.73	± 254.90	391.70	* ± 47.44
0.3	674.24	± 133.83	444.63	± 52.48
1	539.43	± 133.35	565.41	± 72.35
3	1056.66	± 503.34	1035.37	± 366.46
10	874.02	± 321.01	713.63	± 236.11
30	851.91	± 206.72	601.76	± 170.57
100	957.68	± 115.54	903.12	± 104.11
300	468.44	± 63.53	766.92	± 144.60
1000	761.81	± 330.15	1133.05	± 148.23

Table displays desensitization time constants for each GABA concentration in the experimental groups * *p* < 0.001.

## Supplementary Materials

The following are available online at http://www.mdpi.com/2076-3425/8/12/217/s1, Figure S1: Capacitance histogram, Figure S2: Reversal potential for GABA in recorded MSNs, Figure S3: Dose response of GABA in cholinergic interneurons.
