# Supplementary material for: Striatal Neurodegeneration that Mimics Huntington’s Disease Modifies GABA-induced Currents
Source: Brain Sci. 2018 Dec 6;8(12):217. doi: 10.3390/brainsci8120217 (PMC6316731; doi:10.3390/brainsci8120217)
Supplement: Supplementary file 1 [file brainsci-08-00217-s001.pdf]

Supplementary Materials

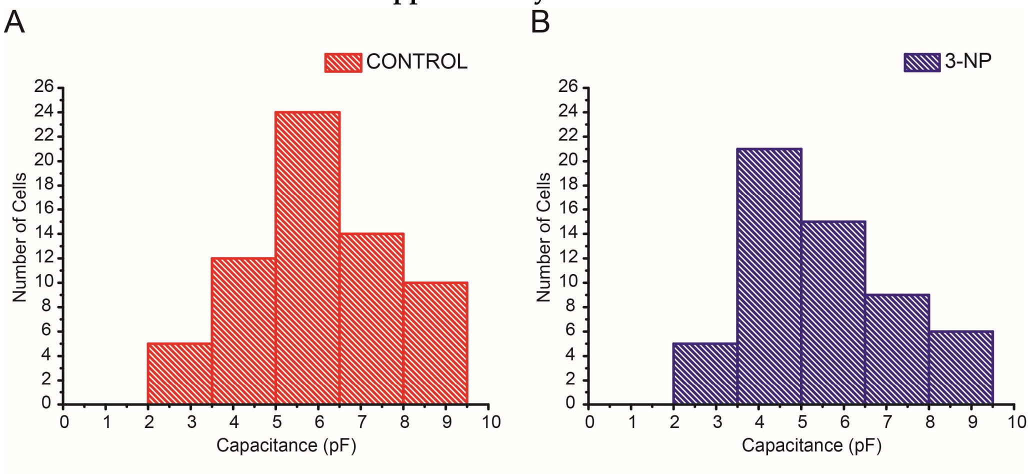

Figure S1. Capacitance histogram. (**A**) Frequency histogram for neurons in the control group. The average capacitance was 6 ± 0.20 pF, (n = 66). (**B**) Frequency histogram for neurons in the group treated with 3-NP. Capacitance average was 5.53 ± 0.25 pF, (n = 59)

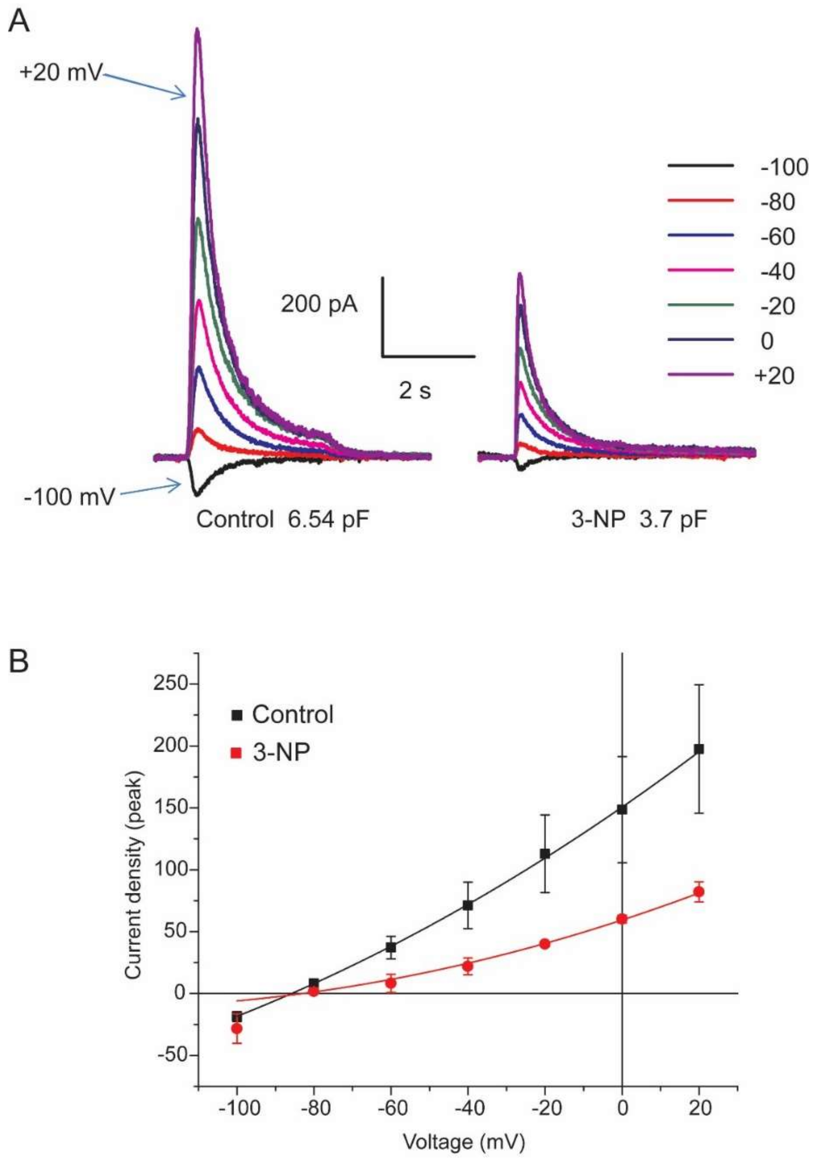

Figure S2. Reversal potential for GABA in recorded MSNs. (**A**) Representation of the current-voltage traces in a control group neuron and in a 3-NP group neuron. The current was generated by applying 100 μM GABA. The voltages used to generate the currents ranged from −100 mV to +20 mV in voltage steps of 20 mV. Plotted traces are the resulting chloride currents for the different voltages. (**B**) I-V curve of the response **to** GABA in A. A second-order polynomial was fit to determine the reversal potential in neurons in the control group (−84.89 ± 1.67; n = 10) and neurons in the 3-NP group (−70.87 ± 12.96; n = 10).

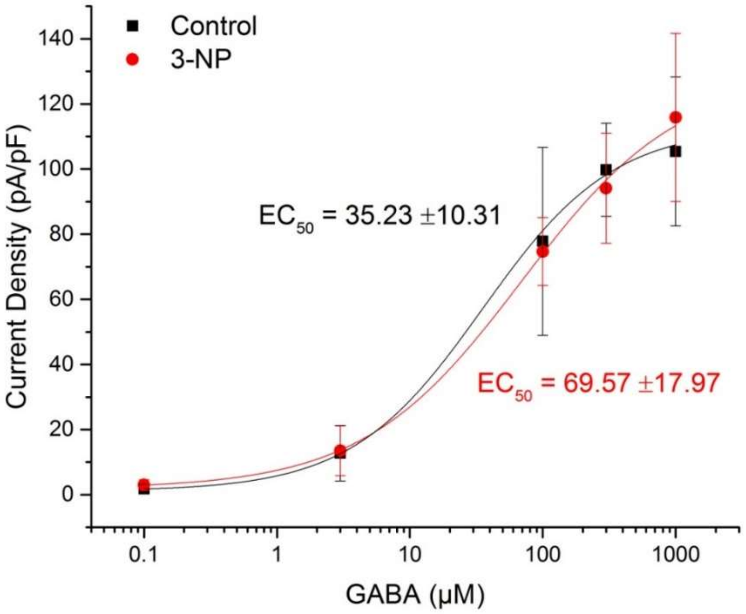

Figure S3. Dose response of GABA in cholinergic interneurons. The following GABA concentrations in both the control group and the 3-NP group were used when recording from cholinergic interneurons: 0.1, 3, 30, 100, 300 and 1000 μM.
